# Great hammerhead sharks swim on their side to reduce transport costs

**DOI:** 10.1038/ncomms12289

**Published:** 2016-07-26

**Authors:** Nicholas L. Payne, Gil Iosilevskii, Adam Barnett, Chris Fischer, Rachel T. Graham, Adrian C. Gleiss, Yuuki Y. Watanabe

**Affiliations:** 1 National Institute of Polar Research, Tachikawa 190-8518, Japan; 2 Department of Life Sciences, University of Roehampton, London SW15 4JD, UK; 3 Faculty of Aerospace Engineering, Technion, Haifa 32000, Israel; 4 College of Marine and Environmental Sciences, James Cook University, Cairns, Queensland 4878, Australia; 5 Ocearch, Park City, Utah 84068, USA; 6 MarAlliance, San Pedro, PO Box 283, Belize; 7 School of Veterinary and Life Sciences, Centre for Fish and Fisheries Research, Murdoch University, Murdoch, Western Australia 6150, Australia; 8 SOKENDAI (The Graduate University for Advanced Studies), Tachikawa 190-8518, Japan

## Abstract

Animals exhibit various physiological and behavioural strategies for minimizing travel costs. Fins of aquatic animals play key roles in efficient travel and, for sharks, the functions of dorsal and pectoral fins are considered well divided: the former assists propulsion and generates lateral hydrodynamic forces during turns and the latter generates vertical forces that offset sharks' negative buoyancy. Here we show that great hammerhead sharks drastically reconfigure the function of these structures, using an exaggerated dorsal fin to generate lift by swimming rolled on their side. Tagged wild sharks spend up to 90% of time swimming at roll angles between 50° and 75°, and hydrodynamic modelling shows that doing so reduces drag—and in turn, the cost of transport—by around 10% compared with traditional upright swimming. Employment of such a strongly selected feature for such a unique purpose raises interesting questions about evolutionary pathways to hydrodynamic adaptations, and our perception of form and function.

## Abstract (web summary)

Sharks' dorsal fins are thought to assist propulsion and turns while pectoral fins are thought to oppose sharks' negative buoyancy. Here, Payne and colleagues show that hammerhead sharks use an exaggerated dorsal fin to generate lift by swimming on their side.

## Graphical abstract

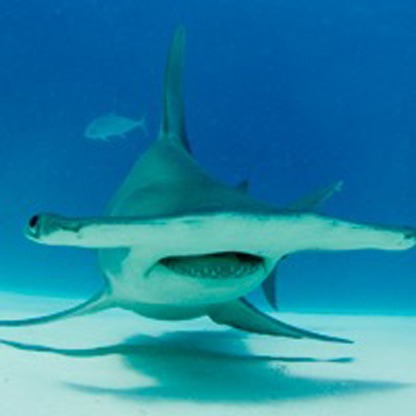

Like other mobile aquatic animals, sharks have evolved a set of morphological traits that facilitate efficient travel in water; a streamlined body shape and assortment of fins are conspicuous examples. Almost all sharks are negatively buoyant^1^,^2^, and use their pectoral fins to generate vertical hydrodynamic force that counteracts gravity^3^,^4^. In contrast, the dorsal fin assists in propulsion^5^ and turning^6^ through the generation of lateral forces^6^,^7^. The prevailing view is that the roles of the pectoral and dorsal fins in sharks are clearly divided in this way.

By measuring body posture of great hammerhead sharks *Sphyrna mokarran* swimming in the wild, we show that this species regularly swims rolled on their side. Because this implies a reconfiguration of the function of their fins during locomotion, we conducted a series of modelling experiments to explore the hydrodynamic consequences of this unusual rolled swimming. Our results show that *S. mokarran* generate lift with their dorsal fin by swimming on their side, and that doing so is a more efficient way to travel than is swimming upright. These findings question the paradigm of the division of labour in shark fins, and highlight that efficient travel is a strong selective agent in driving the evolution of animals.

## Results

### Observations of rolled swimming

**Figure 1:**
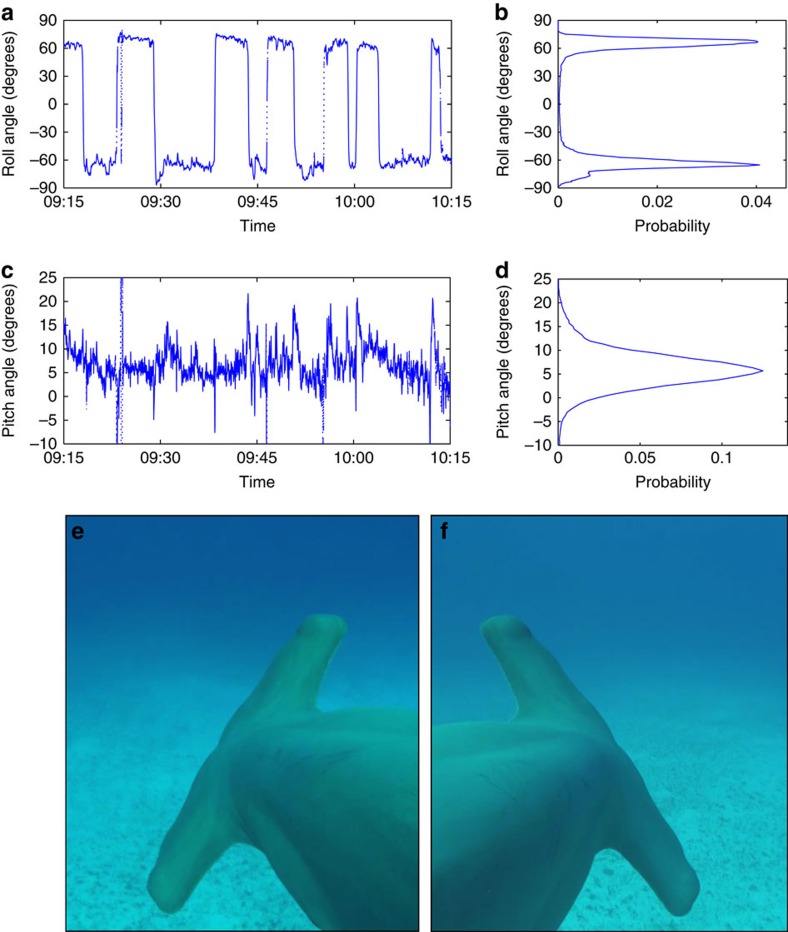
Rolled swimming in great hammerhead sharks *Sphyrna mokarran*. For **a** to **d**, roll and pitch angles were measured by an electronic tag attached to a 295 cm shark's dorsal fin, and monitored as it swam freely at the Great Barrier Reef, northern Australia. (**a**,**c**) A typical hour-long time series for that animal. (**b**,**d**) Probability distributions of roll and pitch angles based on the last 15 h of the monitoring period for the Great Barrier Reef shark. Images in **e**,**f** were taken with a fin-mounted video camera attached to another wild *S. mokarran* (∼350 cm) as it swam rolled to the left and right (respectively) at South Bimini Island, the Bahamas, at absolute roll angles of ∼60° (see Supplementary Movie 2 for examples of this and other wild *S. mokarran* swimming rolled).

We tagged two wild great hammerhead sharks with accelerometer loggers that allow the estimation of body pitch and roll angles as they swim freely in their environment (see Methods, Supplementary Fig. 1 and Supplementary Notes 1 and 2); one at the Great Barrier Reef, Australia, and another off the Mesoamerican Reef, Belize. Unexpectedly, the shark tagged at the Great Barrier Reef spent ∼90% of the 18 h deployment period (which was from early evening till late morning) swimming on its side at absolute roll angles between 50° and 75° (Fig. 1a,b and Supplementary Fig. 2). The shark exhibited this rolling behaviour whether it was ascending, descending or swimming at constant depth, and alternated between rolling to the left and right sides approximately every 5–10 min. An onboard video camera visually confirmed the observations (Supplementary Movie 1). The shark tagged off Belize exhibited a very similar pattern; it was monitored for almost 3 days, and spent the majority of night-time hours swimming at roll angles between 30° and 80°, and tended to swim more upright during daylight hours (Supplementary Fig. 3 and Supplementary Note 2). It is unlikely that this behaviour is a response to the capture, handling and tagging procedure because a further three sharks fitted with onboard video cameras via SCUBA (that is, cameras were fitted to the shark's dorsal fins underwater without being captured or handled) in the Bahamas also exhibited frequent rolled swimming throughout the 2–3 h of each daytime video deployment (Fig. 1e,f and Supplementary Movie 2), and untagged specimens of this species in public aquaria invariably spend a large proportion of time swimming at the same roll angles seen in our wild, tagged animals (Supplementary Movie 3). Ostensibly, this seems a bizarre and unexpected mode of swimming, and has no precedent in the literature. What possible advantage could be obtained by swimming rolled in this way? Doing so would presumably inhibit use of their cephalofoil for detecting electrical signals from benthic prey; therefore, rather than representing a foraging strategy, our hypothesis was that the rolled swimming confers hydrodynamic advantages.

### Hydrodynamics of a swimming shark

Hydrodynamic forces acting on a swimming shark can be conveniently divided into lift *L*, drag *D*, thrust *T* and buoyancy *B*. For simplicity, we will assume that the thrust is generated mainly by the caudal, anal and the second dorsal fins, and is directed along the swimming path, whereas lift and drag are generated by all other fins and by the body of the shark; they are directed perpendicular and parallel to the swimming path, respectively. When swimming at constant speed along a straight horizontal path, all forces cancel out with gravity, *G*:


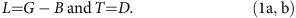


Lift and drag are commonly expressed in terms of the respective coefficients *C*_*L*_ and *C*_*D*_ with


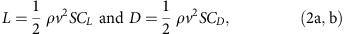


in which *ρ* is the density of water, *v* is swimming speed and *S* is an arbitrary reference area, chosen here as the maximal cross-section area of the body. The lift coefficient depends mainly on the angle between the surface that generates the lift (as a fin) and the swimming direction; the drag coefficient depends mainly on the lift coefficient:


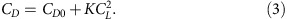


*C*_*D*0_ is the parasite (zero lift) drag coefficient associated with friction between the body and water; *KC*_*L*_^2^ is the induced drag coefficient—the cost of lift generation. At a given speed, the combination of (1a) and (2a) determines the lift coefficient needed to counteract gravity (and hence the set angle of the lift-generating surfaces); the combination of equations (3), (2b) and (1b) determines the thrust needed to maintain that speed.

The induced drag depends on the horizontal span of the lift-generating surfaces, *b*, and on the distribution of lift along these surfaces, reflected in the numerical coefficient *k*_*K*_:


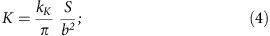


**Figure 2:**
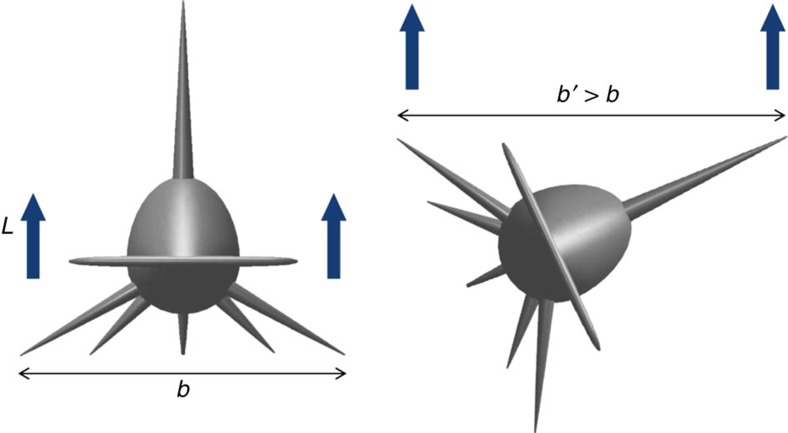
Reconfiguration of lifting surfaces in great hammerhead sharks *S. mokarran*. By swimming rolled, a shark changes the surfaces that generate lift, *L*, from the pair of pectoral fins at zero roll angle (left) to the combination of the pectoral and dorsal fins at greater roll angles (right). For the great hammerhead, doing so increases the effective span of the lifting surfaces, *b*. The model to the right is rolled 65°.

*k*_*K*_ varies between 1.1 and 1.3 for a planar surface^8^. Rolling on its side, a shark gradually transfers some of the lift from its pectoral fins to the dorsal fin (Fig. 2), changing both the horizontal span and the distribution of lift.

Intriguingly, the dorsal fin of a great hammerhead is longer than its pectoral fins; the opposite is true for all other sharks for which we have data (the closely related^9^ scalloped hammerhead *S. lewini* approaches the unique morphology of the great; Supplementary Table 1 and Supplementary Fig. 4). Rolling to its side, a great hammerhead therefore increases the horizontal span of its lift-generating surfaces. Because an increase in horizontal span of lifting surfaces potentially makes the generation of lift more efficient, it is conceivable that great hammerheads induce less drag when they roll to their side than when they swim upright.

**Figure 3:**
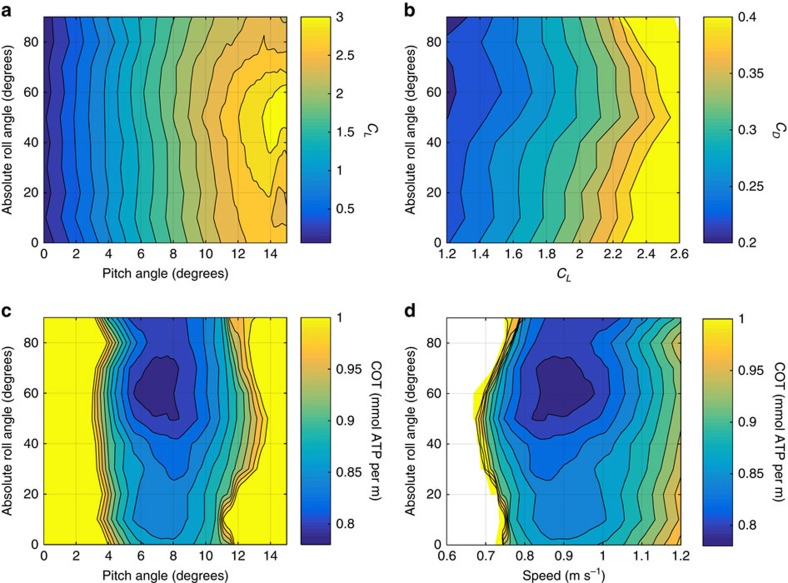
Hydrodynamics of rolled swimming in great hammerhead sharks *S. mokarran*. (**a**) Contours of constant lift *C*_*L*_ and (**b**) drag coefficients *C*_*D*_ for a range of pitch and roll angles, measured through wind tunnel experiments with a physical *S. mokarran* model. (**c**) Contours of constant COT for a 2.95 m shark for a range of roll angle and either pitch angles or (**d**) swimming speeds. COT was estimated from wind tunnel data summarized in **b**, and by assuming values for standard metabolic rate and both chemomechanical and propulsive efficiencies (see Supplementary Notes 4). In **a**, the difference between adjacent contours is 0.2, and in **b–d**, the difference is 0.02.

To examine this possibility, we built a morphologically accurate model of a great hammerhead (see Supplementary Figs 5–7), and conducted a series of experiments in a wind tunnel, keeping the Reynolds number similar to that of a free-swimming shark. In each experiment, the model was set at a constant roll angle (from 0° to 90°, every 10°), and its orientation relative to the flow (equivalent to the pitch angle of a shark swimming at constant depth) was manipulated (between −15° and 15°) while lift and drag were measured with a string balance (see Supplementary Note 3). Remarkably, the minimal drag coefficients occurred at roll angles between 50° and 70° (Fig. 3b), which closely matches the range of roll angles at which our tagged sharks swam in the wild (Fig. 1 and Supplementary Figs 2 and 3). At the relevant range of lift coefficients, the reduction in drag is more than 10% (Fig. 3b). The corresponding energy saving is estimated below.

### Energy savings

Energy expenditure per distance swam (commonly termed the ‘cost of transport', COT) is defined as:


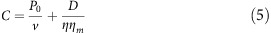


*P*_0_ being the standard metabolic rate, *η* the hydrodynamic propulsion efficiency and *η_m_* the chemomechanical efficiency of the muscles. Given body mass (which we estimated for our Great Barrier Reef shark; see Supplementary Note 4) and temperature (which we measured with our accelerometer loggers), one can estimate the standard metabolic rate^10^, and, assuming the values for *η*, *η_m_* and *B/G* published elsewhere^1^,^11^, the COT follows the data shown in Fig. 3b by equations (1)–(3) and (5), 3, 4, 5. An expanded explanation of calculations for drag, metabolic rate and COT is detailed in Supplementary Notes 4 and 5 and Supplementary Figs 8–12. With all the relevant data listed in Supplementary Table 2, the COT estimates are shown in Fig. 3c,d. Again, displaying remarkable congruence with what the sharks actually do in the wild, COT is minimized at the same roll and pitch angles (between 50° and 70°, and 6° and 8°, respectively), and at the same speeds (between ∼0.8 and 1.0 m s^−1^) exhibited by the wild sharks (Fig. 1 and Supplementary Figs 2 and 3). The gains are significant; ∼8% less energy is used to travel a given distance when swimming rolled than when swimming upright (0.8 versus 0.86 mmol ATP per m; Fig. 3c,d).

## Discussion

Like many other aquatic animals, great hammerhead sharks have evolved morphological traits that facilitate efficient travel. However, unique among species possessing a dorsal fin, great hammerheads employ a drastic reconfiguration of its traditional role in locomotion. Great hammerheads are also one of the most recently diverged of all shark species (∼5 million years ago^9^); therefore, in the context of 450 millions years of chondrichthyan evolution, this solution to minimizing travel costs is relatively new. The variable efficiencies of lift generation among other negatively buoyant fish principally arises from the variable pectoral fin morphologies^12^; the blue shark *Prionace glauca*, which has long and narrow pectoral fins, exemplifies selection of this trait. It is therefore curious that the great hammerhead shark has taken such a different route to evolving lifting surfaces.

Hammerheads possess a number of morphological innovations related to their sensory capacity and manoeuvrability: greater lateral flexture of the body and tight turning capacity^13^,^14^ appear critical to the foraging behaviour of this group that is also related to their unique cranial morphology. These hunting requirements in turn may select for enlargement of the dorsal fin to generate the required lateral forces for performing such manoeuvres. Our work provides an interesting example of how the evolution of novel morphological characteristics for the purpose of one behaviour can result in a drastic shift in the function of existing morphology. It also further highlights that efficient travel is a strong selective agent in driving the evolution of organisms^15^, in particular those facing substantial costs for movement, such as perpetually active aquatic animals. Understanding how animals reduce the effects of drag on their mobility is an important area of research, not just for zoologists, but also mechanical engineers striving to find biomimetic solutions for man-made designs, and even olympic swimmers trying to break world records (the ‘fish kick' stroke, where submerged swimmers swim rolled on their side, revolutionized competitive swimming). With most fully aquatic animals difficult to observe in nature, our work highlights bio-logging technology's important role in revealing novel hydrodynamic adaptations that change our perception of form and function.

## Methods

### Accelerometer and video data collected from wild sharks

For accelerometer deployments, both sharks were captured by fishing and were fitted with tri-axial accelerometer loggers attached to the dorsal fin using established methods^16^. The 295 cm (total length) female shark captured at the Great Barrier Reef was fitted with a Little Leonardo video camera and PD3GT logger (maximum dimensions 150 × 70 × 30 mm, 260 g in air) that recorded acceleration at 16 Hz and both swim speed, depth and temperature at 1 Hz, and it detached from the shark ∼18 h after tagging. Only the last 15 h were used for analysis. The 273 cm male shark captured at the Mesoamerican Barrier Reef near Lighthouse Reef Atoll, Belize was fitted with a ‘daily-diary' (ref. 17; maximum dimensions 150 × 50 × 35 mm, 260 g in air; Supplementary Fig. 1), which recorded tri-axial acceleration, depth and temperature at 8 Hz. The package detached from the shark 66 h following tagging. Both packages were recovered using very high frequency (VHF) telemetry. Analysis and results are detailed in Supplementary Notes 1 and 2 and Supplementary Figs 2 and 3. For the three female sharks (∼250, 300 and 350 cm total length) fitted with video cameras in the Bahamas (throughout January to February 2016 at South Bimini Island), each shark was approached underwater by a SCUBA diver, and a miniaturized (71 × 71 × 39 mm, 152 g in air) video camera (GoPro Hero4) was attached to the anterior edge of the dorsal fin with a double-armed clamp as the shark swam by. Video cameras automatically detached from the sharks after 3 h and the footage was examined for evidence of rolled swimming. Examples of rolled swimming in these sharks are shown in Supplementary Movie 2.

### Wind tunnel experiments

A fifth-scale model of the shark was printed in FullCure720. The general drawing can be found in Supplementary Figs 5 and 6; printer-ready files are available on request. The model had replaceable fins, head and neck. All fins had NACA0015 profile. On the basis of the hypothesis that the caudal, anal and second dorsal fins are used mainly for propulsion and not for the generation of lift, the results presented herein have been measured without them. The experiments were repeated with anal and second dorsal fins attached, and the results remained qualitatively the same (Supplementary Fig. 11). The total length of the model was 640 mm, and the part of the model that went into the tunnel was 431 mm long, ending at the caudal end of the anal and second dorsal fins. Its maximal cross-section area (that was used to obtain the drag and lift coefficients) was 3,870 mm^2^.

The experiments were conducted at the subsonic wind tunnel of the Faculty of Aerospace Engineering, Technion. The wind tunnel is of the open type, with 1 × 1 m square test section, 3 m long. The tunnel is capable of working at 90 m s^−1^. All experiments were conducted at 50 m s^−1^. At this speed, the turbulence intensity is estimated at 0.2%. The Reynolds number based on the total length of the model shark (640 mm) was approximately two million. It matches the Reynolds number of a 3 m shark swimming at 0.7 m s^−1^ in 20° water.

The forces were measured using a six-component string balance and acquired at 5 kHz. The data were low-pass-filtered at 4 Hz, and block-averaged with 500 samples per block. The lift and side force measured during the experiment were of the order of 1 kg; the drag was of the order of 100 g. Measurement accuracy is estimated at 1 g.

### Data Availability

The data that support the findings of this study are available from the corresponding author upon request.

## Additional information

**How to cite this article**: Payne, N. L. *et al*. Great hammerhead sharks swim on their side to reduce transports costs. *Nat. Commun.* 7:12289 doi: 10.1038/ncomms12289 (2016).

## Supplementary Material

Supplementary Information Supplementary Figures 1-12, Supplementary Tables 1-2, Supplementary Notes 1-5 and Supplementary References

Supplementary Movie 1 Shark-mounted video of 295 cm *S. mokarran* swimming at Batt Reef, Queensland, Australia.

Supplementary Movie 2 Shark-mounted video of three *S. mokarran* swimming at South Bimini Island, Bahamas. Video supplied by Andy B. Casagrande IV.

Supplementary Movie 3 Video of two *S. mokarran* swimming rolled in public aquaria in the United States.

## References

[b1] Baldridge D. H. Sinking factors and average densities of Florida sharks as functions of liver buoyancy. Copeia 1970, 744–754 (1970).

[b2] Bone Q. & Roberts B. The density of elasmobranchs. J. Mar. Biol. Assoc. UK 49, 913–937 (1969).

[b3] Harris J. The role of the fins in the equilibrium of the swimming fish: I. Wind-tunnel tests on a model of *Mustelus canis* (Mitchill). J. Exp. Biol. 13, 476–493 (1936).

[b4] Fish F. & Shannahan L. The role of the pectoral fins in body trim of sharks. J. Fish. Biol. 56, 1062–1073 (2000).

[b5] Yates G. T. in Fish Biomechanics (eds. Webb P. W., Weihs D. 177–213Praeger (1983).

[b6] Webb P. Speed, acceleration and manoeuvrability of two teleost fishes. J. Exp. Biol. 102, 115–122 (1983).

[b7] Maia A. & Wilga C. D. Anatomy and muscle activity of the dorsal fins in bamboo sharks and spiny dogfish during turning maneuvers. J. Morphol. 274, 1288–1298 (2013). 23907951 10.1002/jmor.20179

[b8] Raymer D. P. Aircraft Design: a Conceptual Approach 296–298American Institute of Aeronautics and Astronautics (1992).

[b9] Lim D. D., Motta P., Mara K. & Martin A. P. Phylogeny of hammerhead sharks (Family Sphyrnidae) inferred from mitochondrial and nuclear genes. Mol. Phylogenet. Evol. 55, 572–579 (2010). 20138218 10.1016/j.ympev.2010.01.037

[b10] Clarke A. & Johnston N. M. Scaling of metabolic rate with body mass and temperature in teleost fish. J. Anim. Ecol. 68, 893–905 (1999).

[b11] Kushmeri M. J. & Davies R. E. Chemical energetics of muscle contraction. II. Chemistry, efficiency and power of maximally working sartorius muscles. Proc. R Soc. B 174, 315 (1969). 4391323 10.1098/rspb.1969.0096

[b12] Alexander R. M. Principles of Animal Locomotion Princeton University Press (2003).

[b13] Kajiura S. M. & Holland K. N. Electroreception in juvenile scalloped hammerhead and sandbar sharks. J. Exp. Biol. 205, 3609–3621 (2002). 12409487 10.1242/jeb.205.23.3609

[b14] Kajiura S. M., Forni J. B. & Summers A. P. Maneuvering in juvenile carcharhinid and sphyrnid sharks: the role of the hammerhead shark cephalofoil. Zoology 106, 19–28 (2003). 16351888 10.1078/0944-2006-00086

[b15] Gleiss A. C. . Convergent evolution in locomotory patterns of flying and swimming animals. Nat. Commun. 2, 352 (2011). 21673673 10.1038/ncomms1350

[b16] Nakamura I., Watanabe Y. Y., Papastamatiou Y. P., Sato K. & Meyer C. G. Yo-yo vertical movements suggest a foraging strategy for tiger sharks *Galeocerdo cuvier*. Mar. Ecol. Prog. Ser. 424, 237–246 (2011).

[b17] Wilson R. P., Shepard E. L. C. & Liebsch N. Prying into the intimate details of animal lives: use of a daily diary on animals. Endanger. Species Res. 4, 123–137 (2008).

