# Supplementary material for: Great hammerhead sharks swim on their side to reduce transport costs
Source: Nat Commun. 2016 Jul 26;7:12289. doi: 10.1038/ncomms12289 (PMC4963531; doi:10.1038/ncomms12289)
Supplement: Supplementary Information — Supplementary Figures 1-12, Supplementary Tables 1-2, Supplementary Notes 1-5 and Supplementary References [file ncomms12289-s1.pdf]

SUPPLEMENTARY FIGURES

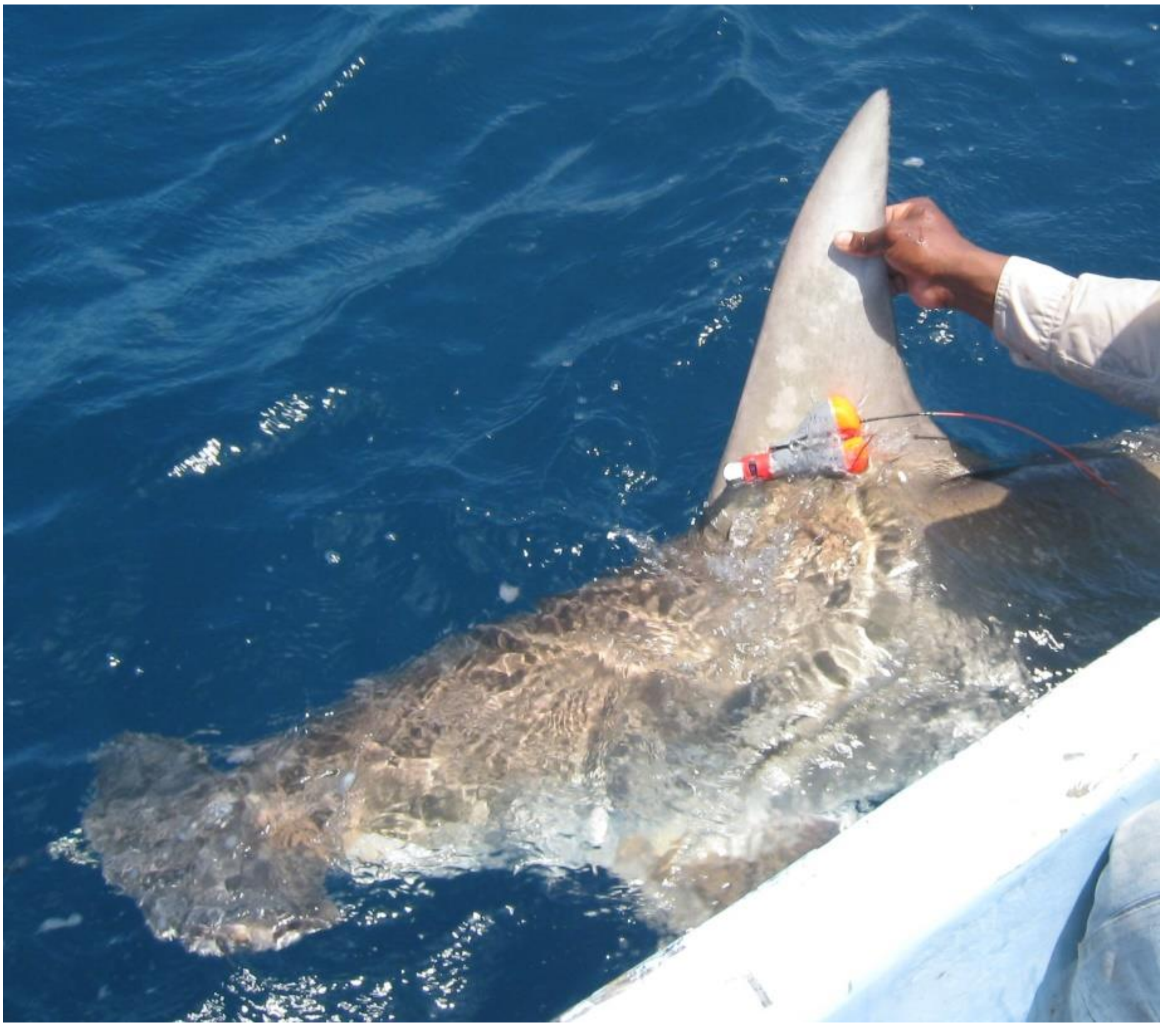

Supplementary Figure 1. Image of an accelerometer package fitted to the 273 cm *S. mokarran* at Mesoamerican Reef, Belize. Image provided by Rachel T. Graham.

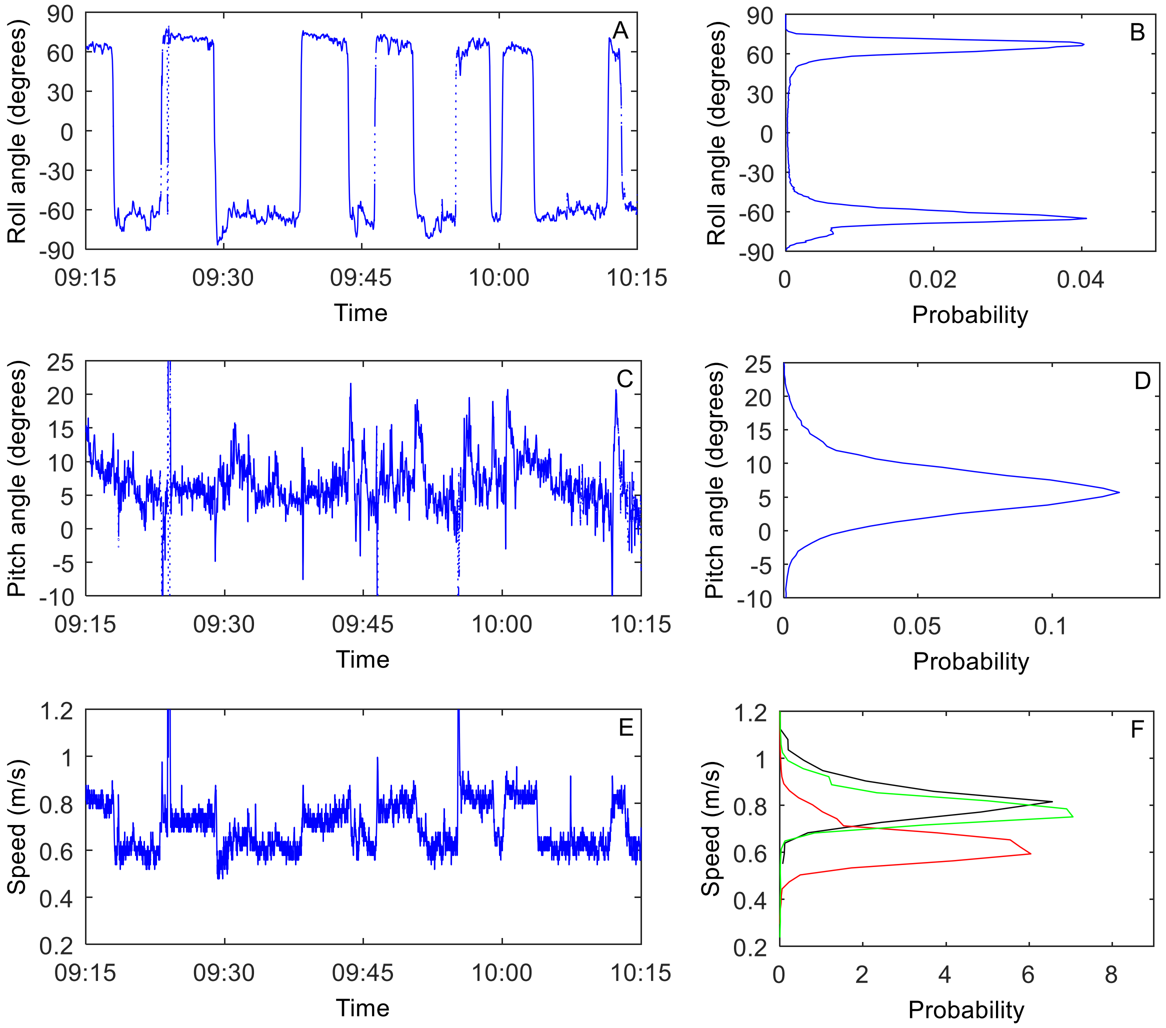

Supplementary Figure 2: One hour sample of the roll angle (a) pitch angle (c) and swimming speed (e) pattern for the 295 cm Batt Reef shark, starting 16 hours after deployment. Panels on the right are summaries of all calculated roll (b) and pitch (d) angles and swimming speeds (f) during the last 15 hours of the 18 hours deployment. In f, the green and red lines represent the speeds when the shark was rolled right and left, respectively (see Supplementary Note 2); the black line represents the speeds when the rolling angle was less than 30 degrees. Only the data for which the total acceleration was less than 0.02g is shown. It comprised 98% percent of the entire data set.

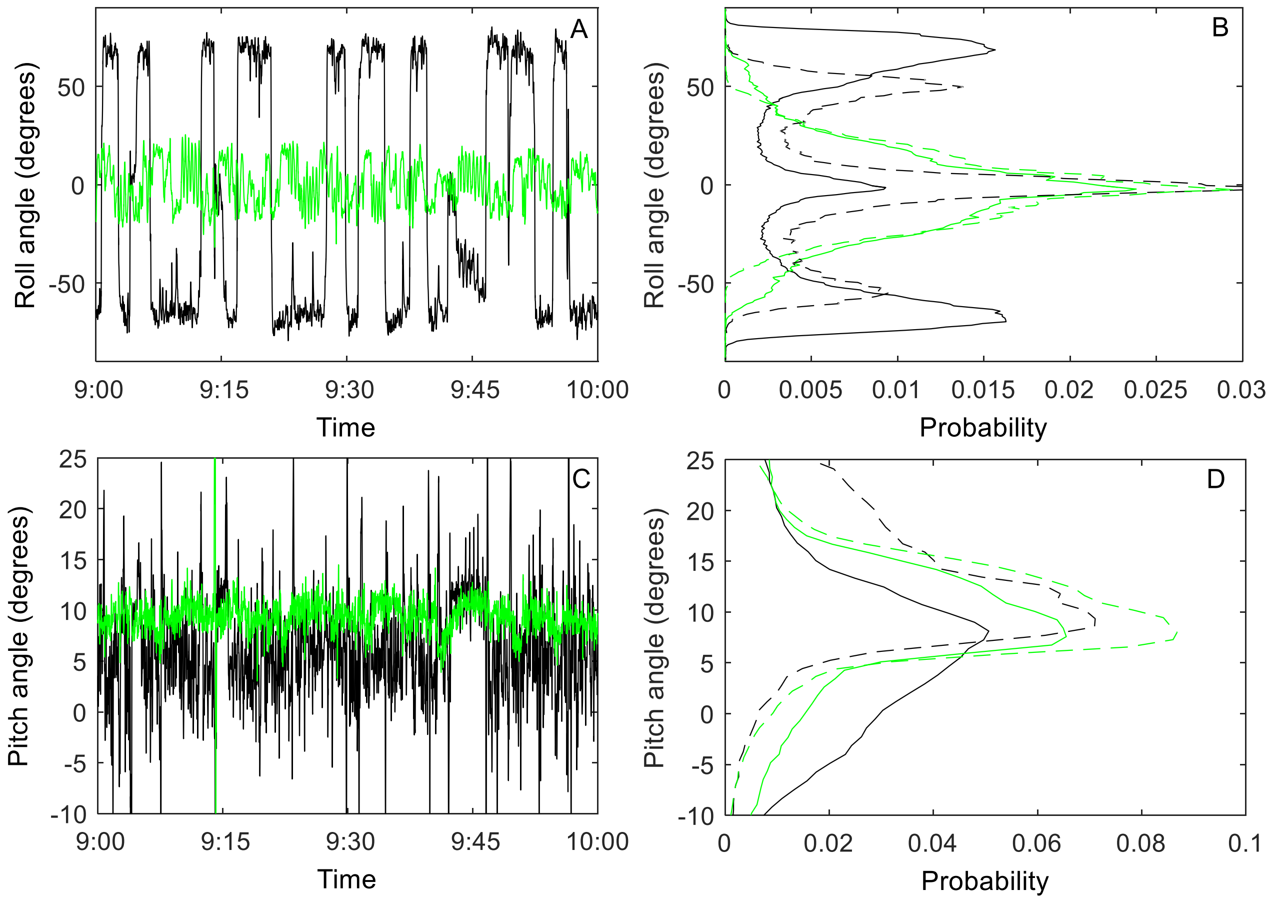

Supplementary Figure 3: Rolled swimming in a 273 cm great hammerhead shark off Belize. (a) a one hour sample of roll and (c) pitch angles during the second evening into the deployment (black line), and at the same time the following morning (green line), with all data used to estimate roll angles. (b) Probability distribution of all calculated roll and (d) pitch angles within the entire deployment period stratified by night (black lines) and day (green lines), and also when all data are used to estimate roll (solid lines) or when absolute acceleration values were below 0.02 g (dashed lines). Because during a large proportion of the high roll angles the total acceleration of the shark was more than 0.02g, the choice of the data set affected the distributions (see Supplementary Note 2). We can only say that this animal was rolled mostly between 30 and 80 degrees during the night, and mostly upright during the day. Its pitch angle was about 8 degrees.

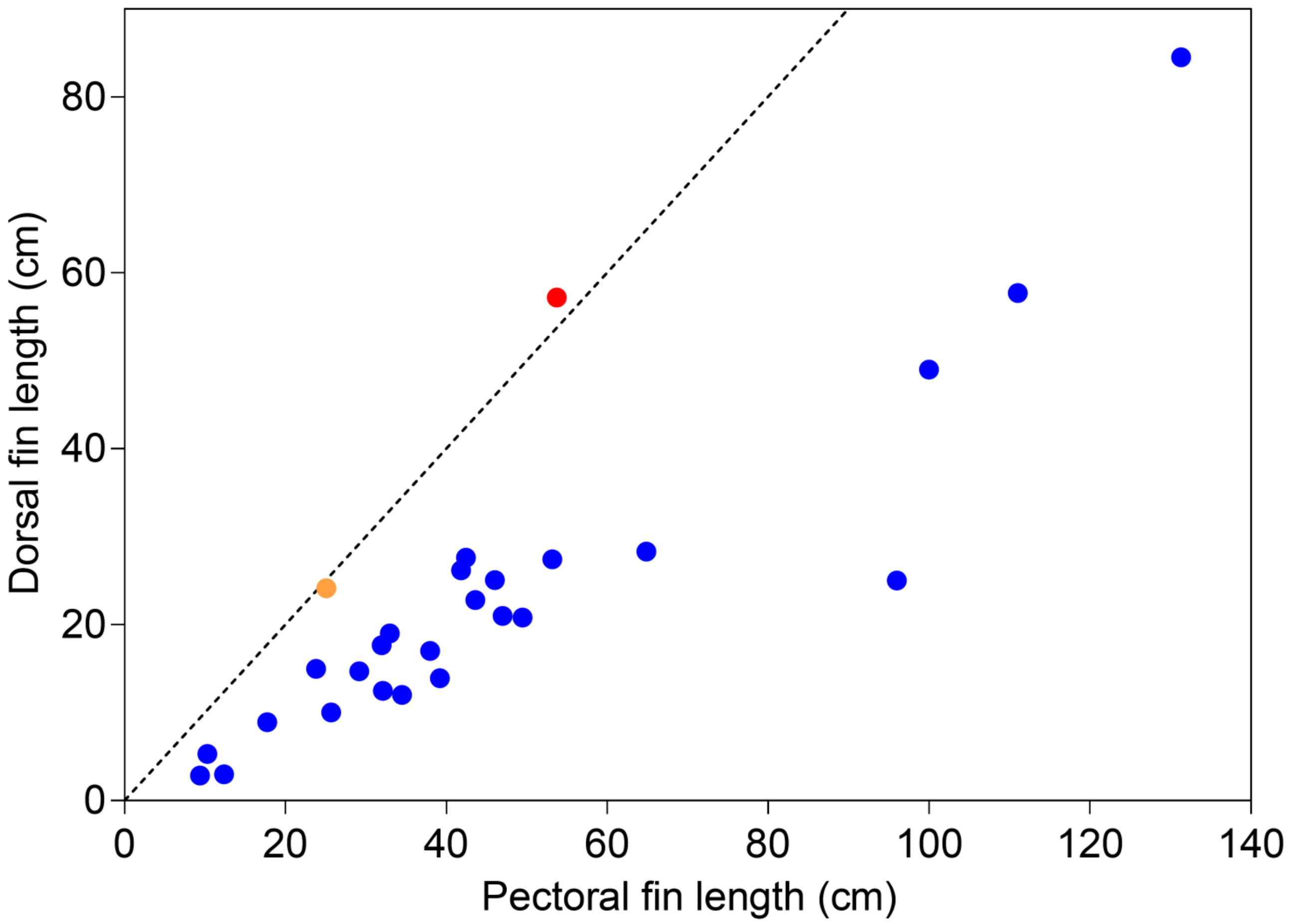

Supplementary Figure 4: Comparison of absolute pectoral and dorsal fin lengths across a broad range of shark species (columns 3 and 4 in Supplementary Table 1). The great hammerhead *S. mokarran* (red datum) is unique in possessing a dorsal fin longer than its pectoral fins (dashed line represents equal lengths); the scalloped hammerhead *S. lewini* (orange datum) comes close.

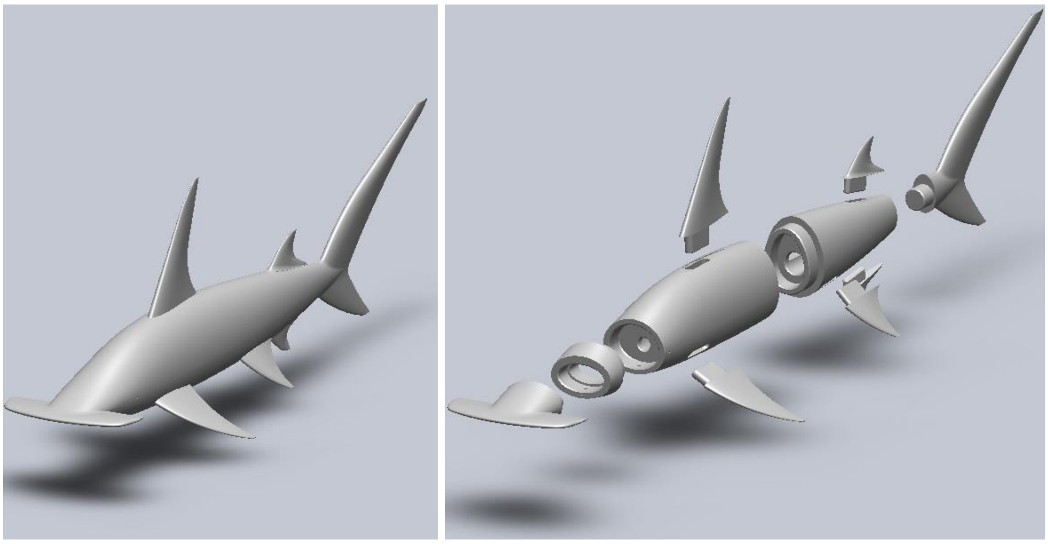

Supplementary Figure 5: The wind tunnel model and its expanded view.

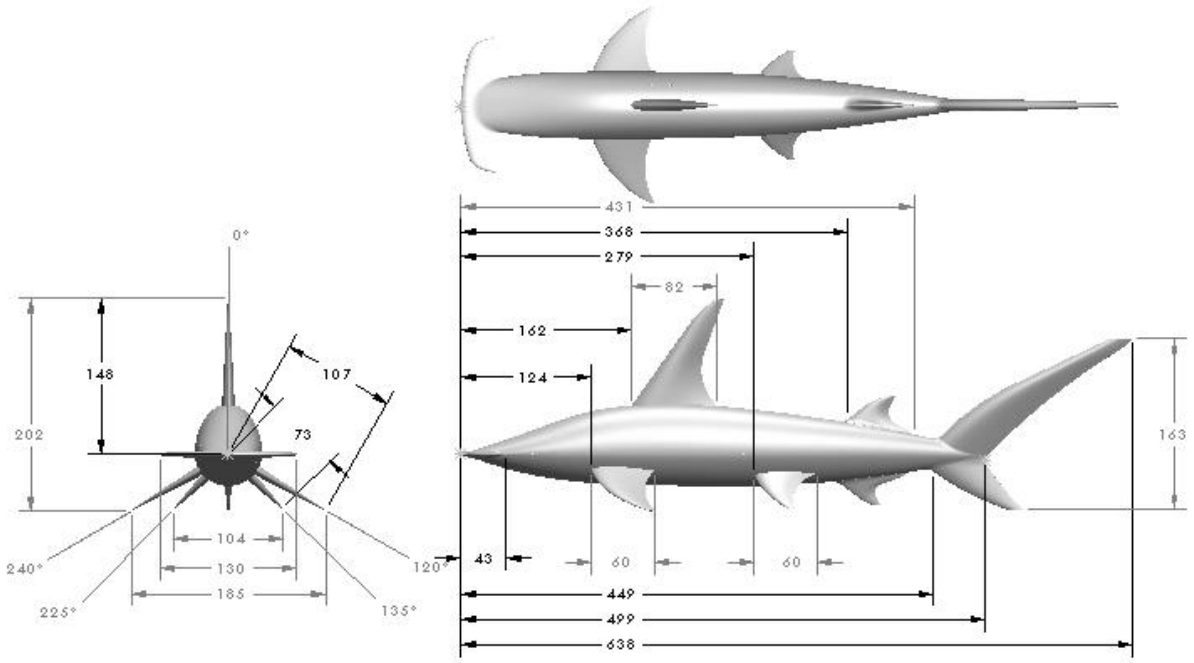

Supplementary Figure 6: Basic dimensions of the model (mm).

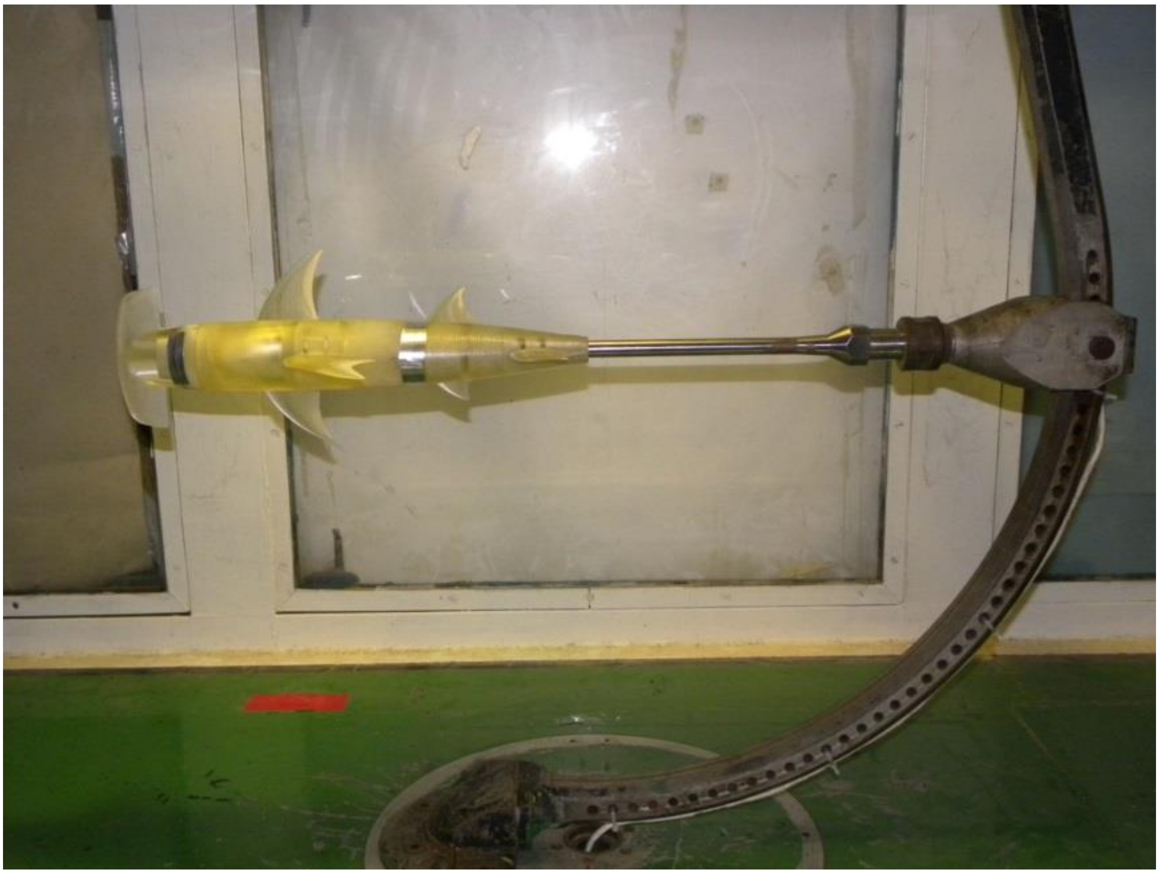

Supplementary Figure 7: Model in the wind tunnel. The flow is from left to right. The equivalent of the sea floor is at the distant wall. The turning table can be seen at the bottom. In this particular experiment, the anal and the second dorsal fins were attached. The aluminum tape across the body serves to secure the connection between the pieces. See Supplementary Note 3 for experimental details. Image provided by Gil Iosilevskii.

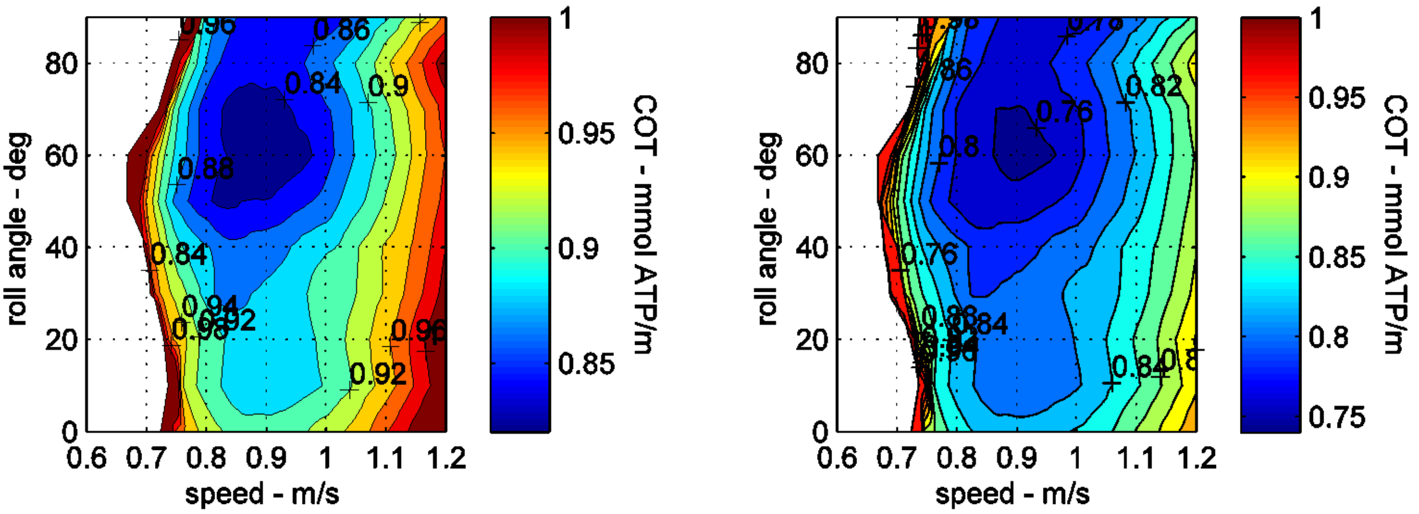

Supplementary Figure 8: Effect of propulsion efficiency, η, on the cost of transport. $\eta = 0.7$ on the left; $\eta = 0.8$ on the right. See Supplementary Note 4 for details.

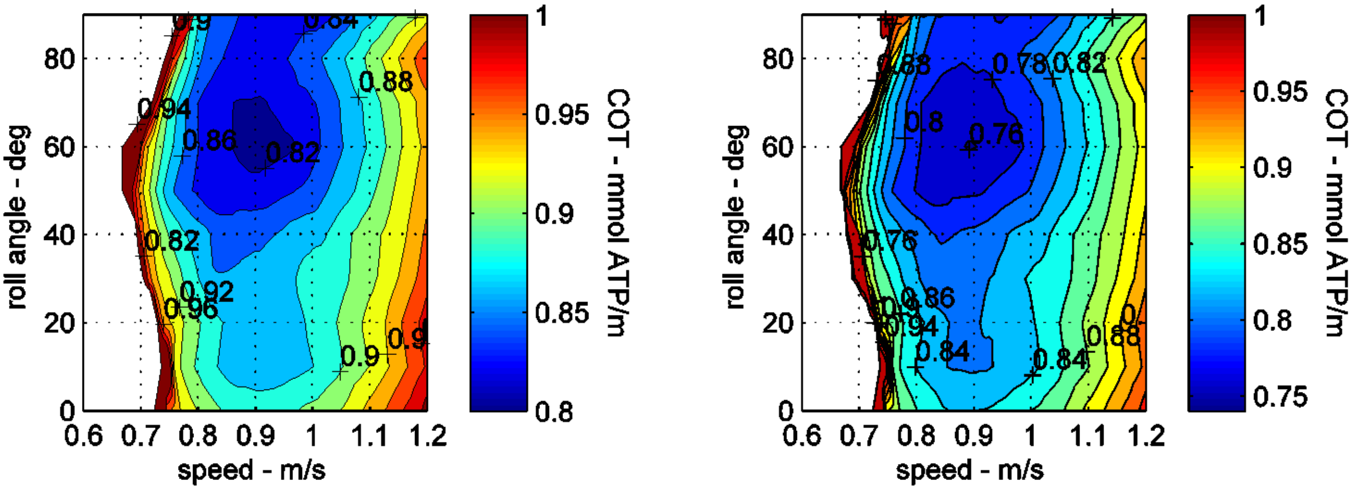

Supplementary Figure 9: Effect of standard metabolic rate on the cost of transport. The nominal rate (equation (S35) in Supplementary Note 4) has been increased by 10% on the left, and reduced by 10% on the right. See Supplementary Note 4 for details.

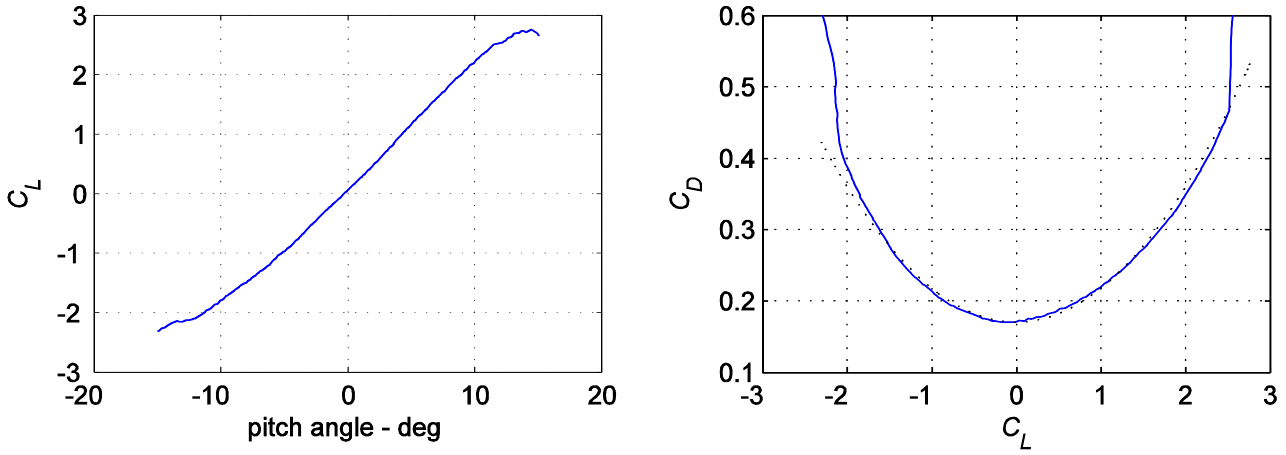

Supplementary Figure 10: Drag and lift coefficients of a model hammerhead at zero roll angle. The reference area is the maximal cross section area of the body. The dotted line on the right figure marks a fitting parabola (equation (S5) in Supplementary Note 5). See Supplementary Note 5 for details.

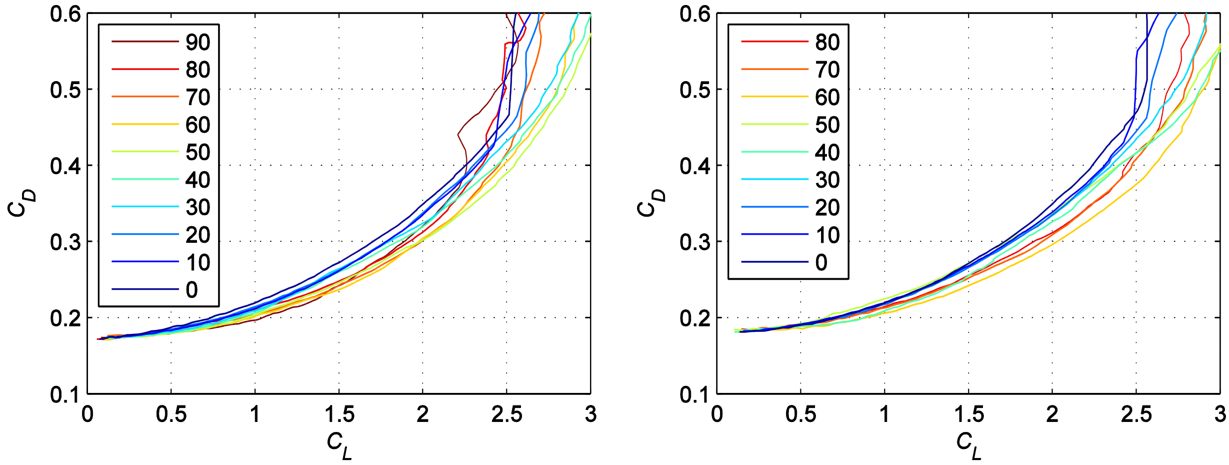

Supplementary Figure 11: Drag versus lift of a model hammerhead at different roll angles (color coded according to the respective legends). The reference area is the maximal cross section area of the body. The difference between the two figures is in the anal and second dorsal fins, which have been removed for the experiments shown on the left. See Supplementary Note 5 for details.

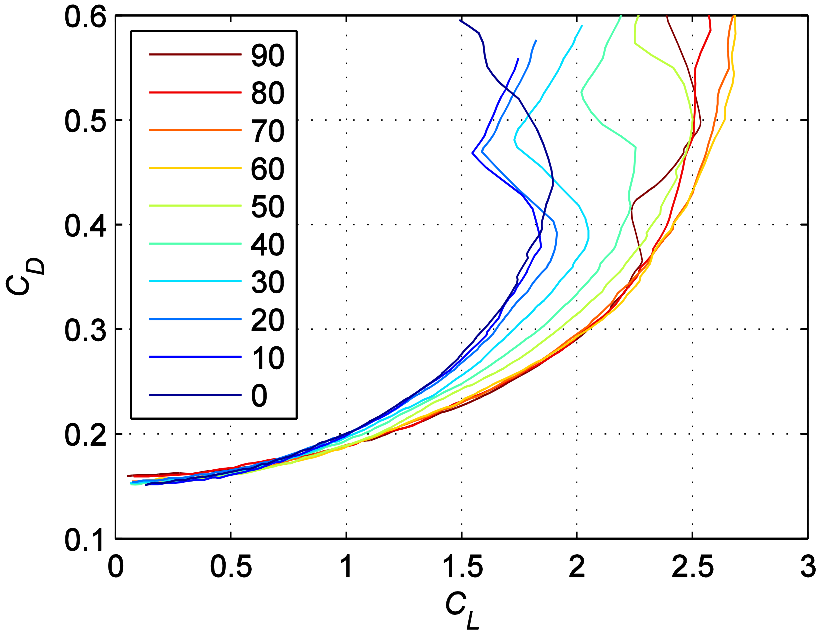

Supplementary Fig. 12: Drag versus lift of a model shark at different roll angles (color coded according to the respective legends). The reference area is the cross section area of the body. In this experiment, the cephalofoil has been replaced by a traditionally-shaped shark head; the anal and the second dorsal fin have been removed. See Supplementary Note 5 for details.

SUPPLEMENTARY TABLES

Supplementary Table 1. Shark fin measurements**.** *Pectoral fin data from Bryan 1972[1] were reported as "length"; all other data are reported as the anterior margin, and all dorsal fin data are presented as height. All lengths are in cm.

Shark species	TL	Pectoral fin	Dorsal fin	Ref.
Broadnose sevengill *Notorynchus cepedianus*	75.3	9.4	2.9	[2]
Little gulper *Centrophorus uyato*	82.0	10.3	5.3	[3]
Wobbegong *Orectolobus leptolineatus*	93.8	17.7	8.9	[4]
Blacktip *Carcharhinus limbatus*	136.0	23.8	15.0	[5]
Grey reef *Carcharhinus amblyrhynchos*	152.5	29.2	14.7	[1]*
Shortfin mako *Isurus oxyrinchus*	179.0	33.0	19.0	[6]
Oceanic whitetip *Carcharhinus longimanus*	190.0	41.8	26.2	[7]
Scalloped hammerhead *Sphyrna lewini*	193.0	25.1	24.1	[5]
Spinner *Carcharhinus brevipinna*	196.0	31.9	17.6	[5]
Blue *Prionace glauca*	196.1	39.2	13.9	[8]
Zebra *Stegostoma fasciatum*	205.0	34.5	12.0	[9]
Sandbar *Carcharhinus plumbeus*	206.0	42.4	27.6	[5]
Galapagos *Carcharhinus galapagensis*	221.8	43.6	22.8	[1]*
Silky *Carcharhinus falciformis*	226.0	47.0	21.0	[1]*
Bull *Carcharhinus leucas*	228.0	46.1	25.1	[5]
Lemon *Negaprion brevirostris*	253.7	49.5	20.8	[5]
False cat *Pseudotriakis microdon*	279.0	25.7	10.0	[10]
Goblin *Mitsukurina owstoni*	312.5	32.1	12.5	[11]
Bluntnose sixgill *Hexanchus griseus*	313.0	38.0	17.0	[1]*
Dusky whaler *Carcharhinus obscurus*	318.0	64.9	28.3	[5]
Tiger *Galeocerdo cuvier*	343.0	53.2	27.4	[5]
Great hammerhead *Sphyrna mokarran*	351.0	53.7	57.2	[5]
Pacific sleeper *Somniosus pacificus*	430.0	12.4	3.0	[12]
Megamouth *Megachasma pelagios*	509.0	96.0	25.0	[13]
Whale *Rhincodon typus*	562.0	100.0	49.0	[14]
Basking *Cetorhinus maximus*	828.5	131.3	84.5	[15]
Great white *Carcharodon carcharias*	na	111.0	57.7	[16]

Supplementary Table 2. Parameter values used to estimate cost of transport.

parameter	value	source
total length	2.95 m	measured
fork length	2.3 m	estimated based on the CAD model
cross section area	0.082 m^2	estimated based on the CAD model
displaced mass	124 kg	estimated based on the CAD model and water density of 1025 kg/m^3
sinking factor	0.048	[17]
standard metabolic rate	0.2274 mmol ATP per s	[18]
propulsion efficiency	0.75	middle of typical range
muscle efficiency	24 Joule per mmol ATP	[19]

SUPPLEMENTARY NOTES

Supplementary Note 1: Using data loggers to determine shark posture relative to earth

The problem

Data loggers were equipped with a three-axis accelerometer that measures the specific force acting on the shark. The precise orientation of the accelerometer relative to the shark is unknown, and we seek the orientation of the shark relative to Earth. For the following analysis, it will be assumed that accelerometer data have been low-pass-filtered to remove the tail-beat frequency (we have used 0.25 Hz), and include only those segments for which the acceleration of the shark was practically zero (we have used a threshold of 0.02g).

Reference frames

We define three orthogonal right-handed reference frames: *A*, *B* and *E*. Frames *A* and *B* are rigidly attached to the shark's body; frame *E* is rigidly attached to Earth. The *x*-axes of all three systems are facing in the general direction of swimming, the *z*-axes are facing down, and the *y*-axes are facing right; when convenient, the axes will be numbered 1, 3 and 2, respectively. The axes of *A* are aligned with the measurement axes of the accelerometer; the *x*- and *z*- axes of *B* coincide with the caudo-cranial and dorso-ventral axes of the body. The *x*- and *y*-axes of *E* are horizontal.

The components of the specific force acting on the shark in the three frames will be denoted $\mathbf{a}^{(A)}$, $\mathbf{a}^{(B)}$ and $\mathbf{a}^{(E)}$. Among the three, $\mathbf{a}^{(A)}$ is known (measured); $\mathbf{a}^{(B)}$ is unknown; $\mathbf{a}^{(E)}$ is assumed known:

$$\tilde{\mathbf{a}}^{(E)} = (0,0,1). \qquad \text{(S1)}$$

The tilde marks here a transpose. Transformation matrices between the reference frames are unknown; in fact, they are the objectives of the data reduction.

Euler rotations

Consider a pair of arbitrary orthogonal reference frames, say, *C* and *D*, sharing a common origin and rotated one relative to the other through angle α about axis $a \in \{1,2,3\}$. The transformation matrix $\mathbf{T}(a,\alpha)$ between *D* and *C*,

$$\mathbf{x}^{(C)} = \mathbf{T}(a,\alpha)\mathbf{x}^{(D)}, \tag{S2}$$

is

$$\mathbf{T}(1,\alpha) = \begin{pmatrix} 1 & 0 & 0 \\ 0 & \cos\alpha & -\sin\alpha \\ 0 & \sin\alpha & \cos\alpha \end{pmatrix}, \quad \mathbf{T}(2,\alpha) = \begin{pmatrix} \cos\alpha & 0 & \sin\alpha \\ 0 & 1 & 0 \\ -\sin\alpha & 0 & \cos\alpha \end{pmatrix}, \quad \mathbf{T}(3,\alpha) = \begin{pmatrix} \cos\alpha & -\sin\alpha & 0 \\ \sin\alpha & \cos\alpha & 0 \\ 0 & 0 & 1 \end{pmatrix}, \tag{S3}$$

depending on the axis about which the rotation took place. **T** has the properties that

$$\mathbf{T}(a,\alpha)\mathbf{T}(a,\beta) = \mathbf{T}(a,\alpha+\beta), \tag{S4}$$

$$\mathbf{T}(a,\alpha)\tilde{\mathbf{T}}(a,\alpha) = \tilde{\mathbf{T}}(a,\alpha)\mathbf{T}(a,\alpha) = \mathbf{I}, \tag{S5}$$

where tilde marks a transpose and **I** is the unit matrix.

Accelerations

We will assume that transformation between frames *E* and *B* can be obtained by a series of two rotations. First, about the *y*-axis through angle θ (pitch), and then about the *x*-axis through angle ϕ (roll):

$$\mathbf{a}^{(E)} = \mathbf{T}(2,\theta)\mathbf{T}(1,\phi)\mathbf{a}^{(B)}, \tag{S6}$$

Equivalently,

$$\mathbf{a}^{(B)} = \tilde{\mathbf{T}}(1,\phi)\tilde{\mathbf{T}}(2,\theta)\mathbf{a}^{(E)} \tag{S7}$$

by (S5). Assuming (S1),

$$\mathbf{a}^{(B)} = \tilde{\mathbf{T}}(1,\phi)\tilde{\mathbf{T}}(2,\theta)\mathbf{a}^{(E)} = \begin{pmatrix} -\sin\theta \\ \cos\theta\sin\phi \\ \cos\theta\cos\phi \end{pmatrix} \tag{S8}$$

by (S3).

Let $\langle\ \rangle$ denote an average of the respective quantity with respect to time. Thus,

$$\langle \mathbf{a}^{(B)} \rangle = \begin{pmatrix} -\langle \sin\theta \rangle \\ \langle \cos\theta \sin\phi \rangle \\ \langle \cos\theta \cos\phi \rangle \end{pmatrix}. \tag{S9}$$

We shall assume that pitch and roll are practically independent; namely

$$\langle f(\theta) g(\phi) \rangle = \langle f(\theta) \rangle \langle g(\phi) \rangle, \tag{S10}$$

for any pair of functions *f* and *g*. We shall also assume that θ is sufficiently small to make the approximation

$$\langle \sin\theta \rangle \langle \cos\theta \rangle \approx \langle \sin\theta \cos\theta \rangle \tag{S11}$$

valid, whereas

$$\langle \sin\phi \rangle = \langle \sin\phi \cos\phi \rangle = 0, \tag{S12}$$

that is, the shark spends the same time rolled right as it spends rolled left. The last assumption will be removed later on. In the interim, we assume that frame *B* is oriented relative to the shark in such a way that (S12) holds.

From (S12),

$$\langle \mathbf{a}^{(B)} \rangle = \begin{pmatrix} -\langle \sin\theta \rangle \\ 0 \\ \langle \cos\theta \cos\phi \rangle \end{pmatrix} \tag{S13}$$

by (S10) and (S9). Now, using (S10) we form

$$\langle \mathbf{a}^{(B)} \tilde{\mathbf{a}}^{(B)} \rangle = \left(\begin{array}{c|c|c} \langle \sin^2\theta \rangle & -\langle \sin\phi \rangle \langle \sin\theta\cos\theta \rangle & -\langle \cos\phi \rangle \langle \sin\theta\cos\theta \rangle \\ \hline -\langle \sin\phi \rangle \langle \sin\theta\cos\theta \rangle & \langle \cos^2\theta \rangle \langle \sin^2\phi \rangle & \langle \cos^2\theta \rangle \langle \sin\phi\cos\phi \rangle \\ \hline -\langle \cos\phi \rangle \langle \sin\theta\cos\theta \rangle & \langle \cos^2\theta \rangle \langle \sin\phi\cos\phi \rangle & \langle \cos^2\theta \rangle \langle \cos^2\phi \rangle \end{array}\right); \tag{S14}$$

from which

$$\langle \mathbf{a}^{(B)} \tilde{\mathbf{a}}^{(B)} \rangle - \langle \mathbf{a}^{(B)} \rangle \langle \tilde{\mathbf{a}}^{(B)} \rangle = \left(\begin{array}{c|c|c} \langle \sin^2\theta \rangle - \langle \sin\theta \rangle^2 & 0 & 0 \\ \hline 0 & \langle \cos^2\theta \rangle \langle \sin^2\phi \rangle & 0 \\ \hline 0 & 0 & \begin{array}{c} \langle \cos^2\theta \rangle \langle \cos^2\phi \rangle \\ -\langle \cos\theta \rangle^2 \langle \cos\phi \rangle^2 \end{array} \end{array}\right) \tag{S15}$$

follows by (S12) and (S11); the off-diagonal terms fall out. In other words, with $\tilde{\mathbf{a}}^{(B)} = \left(a_x^{(B)}, a_y^{(B)}, a_z^{(B)}\right)$,

$$\langle \mathbf{a}^{(B)}\tilde{\mathbf{a}}^{(B)} \rangle - \langle \mathbf{a}^{(B)} \rangle \langle \tilde{\mathbf{a}}^{(B)} \rangle = \begin{pmatrix} \langle a_x^{(B)} a_x^{(B)} \rangle - \langle a_x^{(B)} \rangle^2 & 0 & 0 \\ 0 & \langle a_y^{(B)} a_y^{(B)} \rangle - \langle a_y^{(B)} \rangle^2 & 0 \\ 0 & 0 & \langle a_z^{(B)} a_z^{(B)} \rangle - \langle a_z^{(B)} \rangle^2 \end{pmatrix} \quad \text{(S16)}$$

Transformation between *A* and *B*

Now, the accelerometer is connected to the body at some orientation, represented by a certain transformation matrix $\mathbf{T}_0$. It relates $\mathbf{a}^{(A)}$ and $\mathbf{a}^{(B)}$ by

$$\mathbf{a}^{(B)} = \mathbf{T}_0 \mathbf{a}^{(A)}. \quad \text{(S17)}$$

Consequently,

$$\langle \mathbf{a}^{(B)} \rangle = \mathbf{T}_0 \langle \mathbf{a}^{(A)} \rangle. \quad \text{(S18)}$$

and

$$\langle \mathbf{a}^{(B)}\tilde{\mathbf{a}}^{(B)} \rangle = \mathbf{T}_0 \langle \mathbf{a}^{(A)}\tilde{\mathbf{a}}^{(A)} \rangle \tilde{\mathbf{T}}_0 \quad \text{(S19)}$$

by (S17).

We have already established that $\langle \mathbf{a}^{(B)}\tilde{\mathbf{a}}^{(B)} \rangle - \langle \mathbf{a}^{(B)} \rangle \langle \tilde{\mathbf{a}}^{(B)} \rangle$ has a diagonal form – see (S16) and (S15). The transformation $\mathbf{T}_0\left(\langle \mathbf{a}^{(A)}\tilde{\mathbf{a}}^{(A)} \rangle - \langle \mathbf{a}^{(A)} \rangle \langle \tilde{\mathbf{a}}^{(A)} \rangle\right)\tilde{\mathbf{T}}_0$ that brings the matrix $\langle \mathbf{a}^{(A)}\tilde{\mathbf{a}}^{(A)} \rangle - \langle \mathbf{a}^{(A)} \rangle \langle \tilde{\mathbf{a}}^{(A)} \rangle$ into diagonal form is known as the eigenvalue decomposition. In fact, given $(\lambda_1, \lambda_2, \lambda_3)$, the eigenvalues of $\langle \mathbf{a}^{(A)}\tilde{\mathbf{a}}^{(A)} \rangle - \langle \mathbf{a}^{(A)} \rangle \langle \tilde{\mathbf{a}}^{(A)} \rangle$, and $\mathbf{V}$, the matrix of the respective eigenvectors,

$$\tilde{\mathbf{V}}\left(\langle \mathbf{a}^{(A)}\tilde{\mathbf{a}}^{(A)} \rangle - \langle \mathbf{a}^{(A)} \rangle \langle \tilde{\mathbf{a}}^{(A)} \rangle\right)\mathbf{V} = \tilde{\mathbf{V}} \begin{pmatrix} \lambda_1 & 0 & 0 \\ 0 & \lambda_2 & 0 \\ 0 & 0 & \lambda_3 \end{pmatrix} \mathbf{V} \quad \text{(S20)}$$

by definition, and hence $\mathbf{T}_0 = \tilde{\mathbf{V}}$ and $(\lambda_1, \lambda_2, \lambda_3)$ are the terms on the diagonal in (S16).

In order to obtain the orientation of the accelerometer from $\mathbf{T}_0$ we assume that it is obtained

through a series of three Euler rotations, roll (ϕ_0), pitch (θ_0) and yaw (ψ_0), in that order. In this case,

$$\mathbf{T}_0 = \mathbf{T}(1,\phi_0)\mathbf{T}(2,\theta_0)\mathbf{T}(3,\psi_0) =$$

$$= \left(\begin{array}{c|c|c} \cos\theta_0 \cos\psi_0 & -\cos\theta_0 \sin\psi_0 & \sin\theta_0 \\ \hline \cos\psi_0 \sin\theta_0 \sin\phi_0 + \sin\psi_0 \cos\phi_0 & -\sin\psi_0 \sin\theta_0 \sin\phi_0 + \cos\psi_0 \cos\phi_0 & -\cos\theta_0 \sin\phi_0 \\ \hline -\cos\psi_0 \sin\theta_0 \cos\phi_0 + \sin\psi_0 \sin\phi_0 & \sin\psi_0 \sin\theta_0 \cos\phi_0 + \cos\psi_0 \cos\phi_0 & \cos\theta_0 \cos\phi_0 \end{array} \right) \tag{S21}$$

and ϕ_0, θ_0 and ψ_0 can be recovered from $\mathbf{T}_0$ with

$$\tan\psi_0 = -\frac{[\mathbf{T}_0]_{12}}{[\mathbf{T}_0]_{11}}, \; \sin\theta_0 = [\mathbf{T}_0]_{13}, \; \tan\phi_0 = -\frac{[\mathbf{T}_0]_{23}}{[\mathbf{T}_0]_{33}}. \tag{S22}$$

Combining (S7), (S17) and (S21), one will find

$$\mathbf{a}^{(E)} = \mathbf{T}(2,\theta)\mathbf{T}(1,\phi)\mathbf{a}^{(B)} = \mathbf{T}(2,\theta)\mathbf{T}(1,\phi)\mathbf{T}(1,\phi_0)\mathbf{T}(2,\theta_0)\mathbf{T}(3,\psi_0)\langle\mathbf{a}^{(A)}\rangle$$

$$= \mathbf{T}(2,\theta)\mathbf{T}(1,\phi+\phi_0)\mathbf{T}(2,\theta_0)\mathbf{T}(3,\psi_0)\langle\mathbf{a}^{(A)}\rangle. \tag{S23}$$

In other words, the roll angle between *B* and *A* systems actually reflects the average roll angle and may not truly represent the rotation of the accelerometer relative to the body. If the shark has no preference angle, ϕ_0 can be interpreted as the angle between the accelerometer and the body.

Transformation between *B* and *E*

Given $\mathbf{T}_0$, the components of the specific force in the body reference frame, $\mathbf{a}^{(B)} = \mathbf{T}_0\mathbf{a}^{(A)}$, follow by (S17), and with $\tilde{\mathbf{a}}^{(B)} = \left(a_x^{(B)}, a_y^{(B)}, a_z^{(B)}\right)$, the angles of the shark relative to Earth, θ and ϕ, follow with

$$\sin\theta = -a_x^{(B)}, \quad \tan\phi = a_y^{(B)} \big/ a_z^{(B)} \tag{S24}$$

by (S8).

Supplementary Note 2: Accelerometer data handling

The Batt Reef data logger included a three-axis accelerometer, speed sensor, and depth and temperature sensors. The former was sampled at 16 Hz; the latter two were sampled at 1 Hz. The Belize data logger (shown in Supplementary Fig. 1) included a three-axis accelerometer and depth and temperature sensors, with all three variables sampled at 8 Hz. The first three hours were discarded to minimize capture artefacts, leaving approximately 15 and 63 hours of data for the Batt Reef and Belize sharks, respectively.

Accelerometer data were low-pass filtered at 0.25 Hz to remove acceleration signals caused by the tail-beat frequency at 0.4 Hz. The data were sorted, removing segments for which total acceleration of the shark exceeded 0.02g; 98% and 46% of the data remained for the Batt Reef and Belize sharks, respectively. Orientation of the accelerometer relative to the shark (13 and 13 degrees left roll, 9 and 22 degrees pitch down and 18 and 4 degrees left yaw for the Batt Reef and Belize sharks, respectively), and orientation of the shark relative to Earth (Fig. 1 main document; Supplementary Fig. 3) were found using the paradigm described in Supplementary Note 1. Whereas the orientation angles of the Batt Reef shark could be estimated with confidence for almost the entire deployment period (98% of records), total accelerations produced by the Belize logger were often high after low-pass filtering, so roll angles could be estimated with a high degree of confidence for just under half of the accelerometer records. We therefore presented estimates of roll and pitch angles calculated from the reduced dataset (i.e. where total shark acceleration was less than 0.02g) and also from the entire dataset (i.e. no acceleration threshold) for the Belize shark. Note that pitch angle of the shark – measured between its caudo-cranial axis and the horizon - is a sum of its angle of attack (the angle between is caudo-cranial axis and the direction of swimming) and its trajectory angle (the angle between the direction of swimming and the horizon). The first constituent is akin to the pitch angle measured in the wind tunnel, and is essentially determined by the swimming speed; the second constituent reflects the yo-yo motion of the shark. Because the depth of the shark after many hours of swimming remained within a few tens of meters from its

initial depth, the average of the second constituent is zero, and therefore the most probable pitch angle reflects the average of the angle of attack, which is comparable with the wind tunnel measurements.

Data from the speed sensor for the Batt Reef shark are shown in Supplementary Fig. 2. There is a clear correlation between speed and roll angle of the shark, with lower readings when the shark was rolled left. It is plausible that under these conditions the speed sensor was in the leeward shadow of the dorsal fin. Because the readings of the sensor were similar when swimming upright or rolled right, we deem 0.8 m/s as the average swimming speed during the deployment.

Supplementary Note 3: Wind tunnel experiments

The wind tunnel

The experiments were conducted at the subsonic wind tunnel of the Faculty of Aerospace Engineering, Technion. The wind tunnel is of the open type, having a 1 by 1 by 3 m test section. The contraction ratio of the inlet is 23. All experiments were conducted at 50 m/s; the Reynolds number based on the total length of the model shark (640 mm) was approximately 2 million. It matches the Reynolds number of a 3 m shark swimming at 0.7 m/s in 20°C water. At 50 m/s, the turbulence intensity across the test section is approximately 0.2%.

The model

A fifths-scale model of the shark was constructed using CAD software based on available statistical data [5] and numerous photographs. It was printed in FullCure720. The general drawing can be found in Supplementary Figs 5 and 6; printer-ready files are available on request. The model had replaceable fins, head and neck. All fins had NACA0015 profile.

The total length of the model was 640 mm. The part of the model that went into the tunnel was 431 mm long, ending at the caudal end of the anal and second dorsal fins. Its maximal cross section area (that was used to obtain the drag and lift coefficients) was 3870 mm^2.

The balance

The model was placed on a six-component string balance. Measurement resolution was 1 μv, which is equivalent to approximately 0.42, 0.36 and 0.26 grams of lift, side-force and drag; the accuracy was about 2 grams in lift and side force, and about 1 gram in drag. The lift and side force measured during the experiment were of the order of 1 kg; the drag was of the order of 100 grams. The data were acquired at 5 KHz. Data were low-pass filtered at 4 Hz, and block averaged with 500 samples per block.

Experiments

In each experiment, the shark was set at a constant bank angle (0,10,…,90 deg) and its orientation relative to the flow (equivalent to the pitch angle of a free swimming shark) was changed between minus 15 and plus 15 degrees, at the rate of approximately 0.5 degree per second. This setup is shown in Supplementary Fig. 7.

Supplementary Note 4: Shark Cost Of Transport

Energy expenditure per distance swam (commonly termed the "cost of transport", COT) is defined as:

$$C = \frac{P_0}{v} + \frac{D}{\eta\eta_m}, \tag{S25}$$

where v is the swimming speed, D is the hydrodynamic drag, P_0 is the standard metabolic rate, η the hydrodynamic propulsion efficiency, and η_m the chemo-mechanical efficiency of the muscles. There are a few intricate details in using this equation to estimate the cost of transport of a 'real' shark from wind-tunnel data. These details are described below.

Lift and drag are commonly expressed in terms of the respective coefficients, C_L and C_D with

$$L = \frac{1}{2}\rho v^2 S C_L, \tag{S26}$$

$$D = \frac{1}{2}\rho v^2 S C_D, \tag{S27}$$

in which ρ is the density of water, and S is an arbitrary reference area, chosen here as the maximal cross section area of the body. The lift coefficient depends mainly on the angle between the surface that generates the lift (as a fin) and the swimming direction; the drag coefficient depends mainly on the lift coefficient (see "Supplementary Note 5" below).

S was correlated with the fork length of the shark, l,

$$S = 0.0155 l^2 \tag{S28}$$

using the 3D CAD model used for the wind tunnel experiments. The fork length of the wind tunnel model was 78% of total length, l_t.

When swimming at constant speed along a straight horizontal path, hydrodynamic lift offsets weight

$$L = W. \tag{S29}$$

The weight of the shark in the water, *W*, can be conveniently expressed in terms of the sinking factor, *β*,

$$W = mg\beta, \tag{S30}$$

in which *m* is the displaced mass of the shark, and *g* is the acceleration of gravity. The sinking factor, *β*, was estimated as 0.048 based on published estimates [17]. The displaced mass of the shark,

$$m = \rho V \tag{S31}$$

has been estimated from its volume *V*, which has been correlated with the fork length *l*,

$$V = 0.0099 l^3 \tag{S32}$$

using the 3D CAD model used for the wind tunnel experiments. Based on this ratio, the displaced mass of a 2.95 m (2.3 m fork length) shark should have been 124 kg if the water density was 1025 kg/m^3. Weight estimates of the fish data base (http://www.fishbase.us/summary/Sphyrna-mokarran.html) range between 122 and 124 kg.

The combination of (S26), (S29) and (S30),

$$C_L = \frac{2mg\beta}{\rho v^2 S}, \tag{S33}$$

determines the lift coefficient needed to offset weight at a given swim speed; the combination of (S26) and (S27),

$$D = mg\beta \frac{C_D}{C_L} \tag{S34}$$

determines drag. The ratio C_D/C_L has been taken from the wind-tunnel experiments and corrected for gill resistance (see "Supplementary Note 5" below).

The propulsion efficiency (η) depends on the swimming gait of a shark. We have no simple means to estimate it; most studies indicate that its value ranges between 0.7 and 0.8. We have used 0.75, but changing its value within the acceptable range has no qualitative effect on Fig. 3c-d in the main document (Supplementary Fig. 8).

The muscle efficiency (η_m) depends on the loading and the contraction rate. Again, we have no simple means to estimate it, and therefore we took a constant 24 Joule per mmol ATP after Kushmerick and Davies [19].

There is no consensus about the standard metabolic rate (P_0) of large sharks[20]. In this study it was estimated with

$$P_0 = 0.0193\left(m(1+\beta)\right)^{0.8} \exp\left(15.7 - \frac{5020}{\tau}\right)$$

$$= 0.226\left(\frac{m(1+\beta)}{130}\right)^{0.8} \exp\left(\frac{5020}{293} - \frac{5020}{\tau}\right) \qquad \text{(S35)}$$

mmol ATP per second, where τ is the (absolute) body temperature [18] and m is measured in kg. The results shown in the text are for τ = 293 °K. Changing the standard metabolic rate by 20% has no qualitative effect on the conclusions of the paper (Supplementary Fig. 9).

Supplementary Note 5: Drag

Theory

Hydrodynamic forces acting on a swimming shark can be conveniently (albeit somewhat ambiguously) divided into lift *L*, drag *D*, thrust *T* and buoyancy *B*. For simplicity, we will assume that the thrust is generated mainly by the caudal, anal and the second dorsal fins, and is directed along the swimming path; whereas lift and drag are generated by all other fins and by the body of the shark, they are directed perpendicular and parallel to the swimming path, respectively. When swimming at constant speed along a straight horizontal path, all forces cancel out with gravity, *G*:

$$L = G - B, \tag{S1}$$

$$T = D. \tag{S2}$$

Lift and drag are commonly expressed in terms of the respective coefficients, C_L and C_D with

$$L = \frac{1}{2}\rho v^2 S C_L, \tag{S3}$$

$$D = \frac{1}{2}\rho v^2 S C_D, \tag{S4}$$

in which ρ is the density of water, and *S* is an arbitrary reference area, chosen here as the maximal cross section area of the body. The lift coefficient depends mainly on the angle between the surface that generates the lift (as a fin) and the swimming direction; the drag coefficient depends mainly on the lift coefficient. It is commonly approximated by a parabola:

$$C_D = C_{D0} + K C_L^2. \tag{S5}$$

C_{D0} is the parasite (zero lift) drag coefficient, associated with friction between the body and water; KC_L^2 is the induced drag coefficient – the cost of lift generation. An example can be found in Supplementary Fig. 10.

The weight of the shark in water, $G - B$, is commonly expressed in terms of the sinking factor, *β*,

$$G - B = mg\beta, \tag{S6}$$

in which m is the displaced mass of the shark, and g is the acceleration of gravity.

At a given swimming speed, the combination of (S29), (S26) and (S30),

$$C_L = \frac{2mg\beta}{\rho v^2 S}, \tag{S7}$$

determines the lift coefficient needed to counteract gravity; the combination of (S2), (S27) and (S5),

$$T = \frac{1}{2}\rho v^2 S C_D, \tag{S8}$$

determines the thrust needed to maintain that speed. In turn, the lift coefficient determines the angle between the lift generating surfaces and the flow.

The induced drag depends on the horizontal span of the lift generating surfaces, b, and on the distribution of lift along these surfaces, reflected in the numerical coefficient k_K:

$$K = \frac{k_K}{\pi}\frac{S}{b^2}; \tag{S9}$$

k_K varies between 1.1 and 1.3 for a planar surface [21]. Rolling on its side, a shark gradually transfers some of the lift from its pectoral fins to the dorsal fin, changing both the horizontal span b and the distribution of lift, reflected in k_K. The effect of roll is shown in Supplementary Fig. 11 and recomplied in Fig. 3b in the text.

Experiments: the effect of configuration

We believe that the purpose of the second dorsal and the anal fins is to facilitate propulsion. Consequently, the drag measurements that appear in the text were made with the fins removed. Restoring the fins has no qualitative effect on the results, as shown on the right plate in Supplementary Fig. 11.

We also examined the influence of the cephalofoil on the results, by replacing it with a traditionally-shaped shark head. The results remained qualitatively the same. They are shown in Supplementary Fig. 12.

Corrections

In conducting the experiments, we were careful to preserve the similarity in the Reynolds number, which affects both the parasite drag coefficient C_{D0} and the lift coefficient at which the flow separates from the lifting surfaces (approximately 2.5 in Supplementary Fig. 10). Consequently, the drag coefficient could have been used practically 'as is' in estimation of the cost of transport. We did not measure the drag of the caudal fin and the drag of the gills. The former can be accounted for by the hydrodynamic propulsion efficiency. The drag of the gills was accounted for by increasing the measured value of C_{D0} (approximately 0.17) by 0.02.

Supplementary References

1 Bryan, P. G. *The inshore sharks of Guam: Methods of small-boat shark fishing*. (University of Guam, Marine Laboratory, 1972).

2 Sadowsky, V. First record of broad-snouted seven-gilled shark from Cananéia, coast of Brazil. *Boletim do Instituto Oceanográfico de São Paulo* **18**, 33-35 (1970).

3 Wienerroither, R., Bjelland, O., Bachmann, L. & Junge, C. Northernmost record of the little gulper shark *Centrophorus uyato* in the north-eastern Atlantic Ocean, with taxonomical notes on *Centrophorus zeehaani*. *J. Fish. Biol.* **86**, 834-844 (2015).

4 Last, P. R., Pogonoski, J. J. & White, W. T. A new wobbegong shark, *Orectolobus leptolineatus* sp. nov. (Orectolobiformes: Orectolobidae), from the Western Central Pacific. *Descriptions of new sharks and rays from Borneo. CSIRO Marine and Atmospheric Research Paper* **32**, 1-16 (2010).

5 Clark, E. & Von Schmidt, K. Sharks of the central Gulf coast of Florida. *Bull. Mar. Sci.* **15**, 13-83 (1965).

6 Henderson, A. C., Quigley, D. T. & Flannery, K. The shortfin mako shark *Isurus oxyrinchus* Rafinesque, and the pelagic stingray *Dasyatis violacea* Bonaparte, in Irish waters. *Ir. Nat J* **26** (1999).

7 Lessa, R., Paglerani, R. & Santana, F. M. Biology and morphometry of the oceanic whitetip shark, *Carcharhinus longimanus* (Carcharhinidae), off north-eastern Brazil. *Cybium* **23**, 353-368 (1999).

8 McKenzie, R. & Tibbo, S. A morphometric description of blue shark (*Prionace glauca*) from Canadian Atlantic waters. *Journal of the Fisheries Board of Canada* **21**, 865-866 (1964).

9 Pillai, S. K. & Kasinathan, C. Note on an oviparous Zebra shark *Stegostoma faciatum* (Hermann) landed at Mandapam. *Journal of the Marine Biological Association of India* **27**, 195-197 (1985).

10 Yano, K. & Musick, J. A. Comparison of morphometrics of Atlantic and Pacific specimens of the false catshark, *Pseudotriakis microdon*, with notes on stomach contents. *Copeia*, 877-886 (1992).

11 Rincon, G., Vaske, T. & Gadig, O. B. Record of the goblin shark *Mitsukurina owstoni* (Chondrichthyes: Lamniformes: Mitsukurinidae) from the south-western Atlantic. *Marine Biodiversity Records* **5**, e44 (2012).

12 Ebert, D., Compagno, L. & Natanson, L. Biological notes on the Pacific sleeper shark, *Somniosus Pacificus* (Chondrichthyes, Squalidae). *California Fish and Game* **73**, 117-123 (1987).

13 Human, B. A., Morrison, S. M. & MacLeod, I. D. Is the megamouth shark susceptible to mega-distortion? Investigating the effects of twenty-two years of fixation and preservation on a large specimen of *Megachasma pelagios* (Chondrichthyes: Megachasmidae). *Records of the Western Australian Museum* **7**, 020 (2012).

14 Silas, E. & Rajagopalan, M. On a recent capture of a whale shark (*Rhincodon typus* Smith) at Tuticorin, with a note on information to be obtained on whale sharks from Indian waters. *Journal of the Marine Biological Association of India* **5**, 153-157 (1963).

15 Springer, S. & Gilbert, P. W. The basking shark, *Cetorhinus maximus*, from Florida and California, with comments on its biology and systematics. *Copeia*, 47-54 (1976).

16 Klimley, A. P. & Ainley, D. G. *Great white sharks: the biology of Carcharodon carcharias*. (Academic Press, 1998).

17 Baldridge, D. H. Sinking factors and average densities of Florida sharks as functions of liver buoyancy. *Copeia* **1970**, 744-754 (1970).

18 Clarke, A. & Johnston, N. M. Scaling of metabolic rate with body mass and temperature in teleost fish. *J. Anim. Ecol.* **68**, 893-905, doi:10.1046/j.1365-2656.1999.00337.x (1999).

19 Kushmerick, M. & Davies, R. The chemical energetics of muscle contraction. II. The chemistry, efficiency and power of maximally working sartorius muscles. *Proc. R. Soc. B.*

174, 315-347 (1969).
20 Payne, N. L. *et al.* A new method for resolving uncertainty of energy requirements in large water-breathers: the 'mega-flume' seagoing swim-tunnel respirometer. *Methods Ecol. Evol.* **6**, 668-677 (2015).
21 Raymer, D. P. *Aircraft design: a conceptual approach*. 296-298 (American Institute of Aeronautics and Astronautics, 1992).
